# Functional basis of primary succession: Traits of the pioneer microbes

**DOI:** 10.1111/1462-2920.16266

**Published:** 2022-11-18

**Authors:** Gaofeng Ni, Rachael Lappan, Marcela Hernández, Talitha Santini, Andrew G. Tomkins, Chris Greening

**Affiliations:** ^1^ Department of Microbiology, Biomedicine Discovery Institute Monash University Clayton Victoria Australia; ^2^ School of Biological Sciences University of East Anglia Norwich Norfolk United Kingdom; ^3^ School of Agriculture and Environment University of Western Australia Perth Western Australia Australia; ^4^ School of Earth, Atmosphere & Environment Monash University Clayton Victoria Australia; ^5^ Securing Antarctica's Environmental Future Monash University Clayton Victoria Australia

## INTRODUCTION

Primary succession describes the establishment of new ecosystems through the colonization of barren substrates (Odum, 1969). Bacteria are typically the first colonizers, which initiate key processes that enable ecosystem establishment and provide resources for fungi, plants, and animals to colonize later. The functional roles of these pioneer microbes are varied; for example, they can weather and detoxify environments to increase habitability, provide organic carbon and bioavailable nitrogen through carbon and nitrogen fixation, and form mutualistic relationships with plant species (Abdulla, 2009; Richardson & Simpson, 2011). As taxonomic and functional diversity increases over annual to decadal scales, communities in successional ecosystems transition from a collection of pioneer microbial species towards a robust and complex ecosystem (Álvarez‐Molina et al., 2012; Brown & Jumpponen, 2015; Frouz et al., 2016). Much literature has explored plant community succession, resulting in a strong understanding of the nature, traits, and interactions of early and late colonizers and the development of strong ecological theory to explain these processes. However, understanding of pioneer microbial communities remains rudimentary, with most literature focusing on taxonomic composition rather than functional traits of colonizing bacteria. Numerous key questions remain, for example: How do the first microbes survive and grow in a barren and often hostile environment with minimal nutrients? How do microbes modify their environment for succeeding generations and species? How do these microbes interact with each other, plants, and animals as the new ecosystem develops? We propose here a series of study systems, methodological approaches, and ecological frameworks to address these questions. In turn, a better understanding of primary succession has broad implications for understanding ecological theory, biogeochemical processes, and responses to climate change. Moreover, by understanding how life colonizes barren and hostile locations, we can better predict how life evolved on Earth and might exist on other planets.

## SYSTEMS AND METHODS FOR STUDYING PRIMARY SUCCESSION

Microbial primary succession occurs in a range of natural, host‐associated, and engineered environments, which yield unique temporal and spatial contexts for the study of pioneer species (Fernández‐Martínez et al., 2017). Glacier forelands, the area of newly exposed land from the leading edge of a retreating glacier, provide a temporal transect (a chronosequence) where the distance from the glacier edge can be used as a proxy for time. Sampling at the glacier edge can provide insight into microbial colonizers of newly exposed terrain, with the progressively older terrain once covered by the glacier harbouring more developed microbial communities and vegetation (Brown & Jumpponen, 2014; Fernández‐Martínez et al., 2017; Strauss et al., 2012; Zumsteg et al., 2012). Volcanic lava flows provide opportunities to study microbial primary succession on truly sterile, naturally occurring substrates. Newly erupted lava crystallizes sterile basalt rock at temperatures of around 1150°C, with little or no organic matter, yet contains metals within minerals that may support initial ecosystem establishment, as well as spatial niches within the physical structure of the rock where endolithic communities may assemble. Human‐engineered sterile environments, such acid mine drainage formed from newly extracted mining waste impose multiple physicochemical pressures that restrict first colonizers to polyextremophiles (Kupka et al., 2007; Liljeqvist et al., 2015; Wierzchos et al., 2018). Host‐associated sterile environments can also provide novel and sometimes medically relevant insights into microbial community development; for example, primary bacterial colonization of the infant human gut results in an oxic environment rapidly becoming anoxic (Palmer et al., 2007), in a manner that varies with mode of delivery and the maternal microbiome (Dominguez‐Bello et al., 2010).

Microorganisms can even colonize extraterrestrial environments. Meteorites, which are also sterile upon entering the Earth's atmosphere, provide extraterrestrial input of minerals that are far from geochemical equilibrium with the geosphere, atmosphere, and hydrosphere (Tait et al., 2017). Some meteorites contain an enormous variety of organic molecules (including amino acids in some cases) that formed over 4.6 billion years ago and have never been exposed to terrestrial life (Schmitt‐Kopplin et al., 2010). Fractures and porosity in the meteorites, and weathering of the external fusion crust over time, again provide ideal niches for microbial colonization and additionally make meteorites ideal microcosms for simulated studies of primary succession. Micrometeorites are far more abundant than meteorites, but geochemically equivalent, and preferentially accumulate in heavy mineral niches in wind‐modified sediments (Tomkins et al., 2016, 2019). Tektites, which are terrestrial ejections of non‐porous natural glass following a meteorite impact, provide a different sterile substrate in which biofilm may be the predominant colonization mechanism. Collectively, these environments provide unique opportunities to study first colonizers and their traits in a range of contexts, including comparisons of the various input sources of microbes and nutrients, and the unique physical and chemical characteristics that shape the developing community.

Integrated methodological approaches are required to gain a mechanistic understanding of primary succession across these diverse ecosystems. Researchers have obtained some understanding of the identity of the first microbial colonizers, for example through marker gene‐based surveys of bacterial, archaeal, and fungal community composition (Zumsteg et al., 2012). However, advances in genome‐resolved metagenomics, multi‐omics, and high‐throughput cultivation mean it is now possible to develop a broader and deeper understanding of the nature, traits, or activities of microorganisms across successional gradients. In turn, these hypothesis‐generating tools can be used to inform ecosystem‐scale biogeochemical, imaging, and tracing experiments, as well as culture‐based physiological and biochemical studies, that will provide a deeper mechanistic understanding of how microorganisms shape their environments and vice versa (Lewis et al., 2021). Landmark examples of integrated methodological approaches include King's studies in the 2000s on succession in volcanic deposits; he combined culture‐dependent microbial genomics, culture‐independent community and enzyme profiling, bulk geochemical analyses, and botanical surveys to demonstrate that trace gas oxidisers are the early colonizers in volcanic deposits and potentially promote plant development (Gomez‐Alvarez et al., 2007; King, 2003; King et al., 2008; King & Weber, 2008). Through integration of cutting‐edge culture‐dependent and culture‐independent approaches, there is now much potential to holistically understand the dynamics of succession and especially how functional traits impact successional trajectories. Each technique is limited in its own capacity, and synergising approaches across different scales facilitates more advanced and comprehensive discoveries (Greening et al., 2019).

## FRAMEWORKS FOR MECHANISTIC UNDERSTANDING OF MICROBIAL SUCCESSION

Mechanistic understanding of the ecological processes shaping microbial colonization in successional ecosystems is minimal. Four processes ultimately determine the composition and function of communities: dispersal, selection, drift, and diversification (Vellend, 2010). Previous work has suggested that the importance of these ecological drivers shifts during successional stages: dispersal dominates during initial stages and selection progressively increases thereafter (Dini‐Andreote et al., 2015). Yet with respect to dispersal, the source of dispersing microorganisms remains undetermined. It is assumed that most microbes that colonize terrestrial substrates, for example volcanic lava flows, mine tailings, and glacier forelands, are dispersed from the atmosphere and sometimes via rainwater. However, this remains to be proven in part due to our limited knowledge of the microbial ecology of the atmosphere (Šantl‐Temkiv et al., 2022). Likewise, although atmospheric dispersal is often thought to be a strictly neutral (stochastic) process, certain microorganisms are likely better adapted to be transported into and survive within the atmosphere (Šantl‐Temkiv et al., 2018). Ultimately, side‐by‐side profiling of the nature, traits, and activities of microorganisms is required in successional ecosystems compared to the surrounding atmosphere and other potential sources (e.g., glacial ice and sub‐glacial soils in glacier forelands). In addition, little is understood about functional traits that dictate whether microorganisms live or die during different successional stages. Strong environmental selection will control initial succession, though this selection can either weaken or strengthen over time depending on whether activities of pioneer microbes generate favourable conditions (e.g., through nitrogen fixation in glacier foreland soils [Strauss et al., 2012]) or unfavourable conditions (e.g., through acidification of mine tailings [Mielke et al., 2003]). Generally, it can be expected that resource competition and other antagonistic interactions will intensify during later successional stages as both microbial community diversity and environmental niche heterogeneity increase, which in turn increasingly favours habitat specialists over generalists.

We predict that the first colonizers in terrestrial environments are self‐sufficient habitat generalists. These microbes will have a range of adaptation strategies that allow them to colonize barren environments: metabolic strategies to acquire nutrients from atmospheric and mineral sources, dormant forms to withstand resource limitation, homeostatic mechanisms to survive physicochemical stresses, and potentially the capacity to mediate adhesion, weathering, and biofilm formation. Recent findings suggest that in oligotrophic ecosystems, trace gas oxidisers are the strongest candidates for the first colonizers (Mielke et al., 2003; Ortiz et al., 2021). As recently reviewed, these bacteria use trace (including sub‐atmospheric) concentrations of hydrogen and carbon monoxide to conserve energy through aerobic respiration and, in some cases, generate biomass through chemosynthetic carbon dioxide fixation (Greening & Grinter, 2022). It is also proposed that metabolic water production by hydrogen oxidisers enhances water availability in desiccated environments (Ortiz et al., 2021). Many of these microbes are also predicted to use the energy derived from trace gas oxidation to acquire other nutrients, including bioavailable nitrogen through nitrogen fixation. Trace gas oxidisers span at least eight different bacterial phyla and are dominant in many extreme environments, including Antarctic desert soils, the aphotic ocean, and acidic mine drainage (Islam et al., 2020; Ji et al., 2017; Martínez‐Pérez et al., 2022). Trace gas oxidisers are also reported to occur in successional volcanic soil ecosystems (Gomez‐Alvarez et al., 2007; Hernández et al., 2020; King, 2003; King & Weber, 2008). Ultimately, by initially relying on energy sources from the ubiquitous atmosphere rather than less accessible minerals embedded in the colonized substrate, pioneer microbes can maintain viability and generate nutrients relatively independently of the physicochemical restraints of the environments they colonize.

Nevertheless, the exact composition and traits of the first colonizers is likely to be modulated by the physical properties, chemical composition, and climatic conditions of the environments colonized. For example, meteorites vary greatly in physicochemical properties, with some being far more reduced than any surface environment on Earth and others being comparatively oxidized and rich in organic compounds. We predict that, whereas meteorites containing only inorganic material will be colonized primarily by gas‐scavenging lithoautotrophs that gradually produce organic carbon, carbonaceous chondrites may also initially sustain lithoheterotrophs and organoheterotrophs that rely on extraterrestrial organic compounds (Abdulla, 2009; Tait et al., 2017).

It can be expected that, over time, microbial communities will increase in biomass and diversity, and become more abundant, diverse, and specialized as other species are recruited. For example, the synthesis of organic carbon by lithoautotrophic trace gas oxidisers on meteorites may stimulate further microbial growth and biofilm formation. Organic carbon in the form of exudates and necromass are favourable carbon and energy sources for succeeding heterotrophic community members. In addition, biofilm‐mediated changes in the surface chemistry or electrochemistry (bioweathering) accelerates the weathering of minerals (Abdulla, 2009; Angell, 1999; Palacios et al., 2019). Such changes may facilitate the succession of more specialist microbes that grow through iron‐ and sulfide‐dependent chemolithoautotrophy. Some metabolically flexible microorganisms may even modulate their energetic strategies during different successional stages; for example, in acid mine drainage, pioneering microorganisms such as *Acidithiobacillus* may shift from an initial hydrogen‐fuelled colonization and acid production towards more specialized iron‐ and sulfide‐based metabolism (Islam et al., 2020; Mielke et al., 2003). In contrast, keystone microbial colonizers in alpine regions or glacier forelands create a more hospitable soil environment, achieved by physically altering the soil texture while chemically transforming major elements into more bioavailable forms (e.g., nitrogen fixation, carbon fixation or the solubilization and mineralization of the soil phosphorus pool) (Richardson & Simpson, 2011). This in turn, can make conditions favourable for the eventual colonization of plant populations, as well as their bacterial and fungal symbionts (Franzetti et al., 2020).

## IMPLICATIONS FOR LIFE ON A CHANGING EARTH AND POTENTIALLY OTHER WORLDS

A more comprehensive understanding of microbial primary succession has significant implications for our insights into and predictions of how ecosystems initially establish, develop, and are altered by climate change. One important implication for the near future is that rapidly expanding glacier forelands, brought about by widespread glacial retreat, create substantial deglaciated areas in which early ecosystems may carry out different biogeochemical functions than older, established soil ecosystems. In the future, this may impact biogeochemical cycling and global biodiversity on an increasingly broad scale (Stibal et al., 2020). Furthermore, climate and microclimate variation, in addition to topographic, fluvial, or aeolian disturbances, may alter the ability of first colonizer species to inhabit new foreland terrains and may drive a shift in the composition of the early ecosystems (Wojcik et al., 2021). Using knowledge of microbial primary succession, we may better predict patterns of new ecosystem establishment and how this might impact global processes.

As primary succession requires microbes to establish a new community under ‘bare minimum’ conditions, their metabolic processes and survival strategies may give some insights into how life evolved on Earth (abiogenesis) and potentially other planets (exobiology). Indeed, the genesis of life on, or transport to, another planet represents primary succession in an extreme context (Michalski et al., 2018; Tomkins et al., 2020). Our understanding of this process on Earth is likely to inform our conceptualisation of the origin of life elsewhere. For example, it has been suggested that abiotic interactions between iron‐containing minerals catalysed the synthesis of organic molecules on the early Earth (White et al., 2015). Furthermore, this research can yield valuable lessons for the biological protection of other planets. Microbial primary colonizers are likely to be highly resilient and adaptable, as they may need to survive atmospheric transport and must establish communities in environments with minimal nutrients: such organisms may be capable of colonizing terrains visited by spacecraft or rovers. For example, halophilic bacteria and archaea may be capable of surviving on Mars by coupling the oxidation of the high concentrations of atmospheric carbon monoxide with the reduction of terrestrial perchlorate (King, 2015). Sterilization procedures may even select for extremophiles that are well suited as primary colonizers (Ghosh et al., 2010). Even terraforming strategies, the practicality and ethics of which have been long debated (McKay et al., 1991), may therefore be informed and potentially mediated by the activities of microbial first colonizers.

## CONCLUSIONS

Much remains uncertain surrounding the functions, metabolic strategies, and impacts of microbial first colonizers in the process of primary succession. Rapid and continuing technological advances in culture‐dependent and culture‐independent methods provide opportunities to integrate techniques and make significant breakthroughs. This will allow scientists to address long‐standing questions surrounding how these organisms survive, how their activities shape their environment to enable colonization by successive organisms, how this varies across different environments, and the original sources of these pioneers. Given most ecological theory of succession developed through studies of plant communities, rather than the earlier microbial pioneers with much broader niche range, there is much potential to test and refine theory. In turn, this may provide clues for how life evolved on Earth and can better inform the search for extraterrestrial life.

## AUTHOR CONTRIBUTIONS

**Gaofeng Ni:** Writing – original draft (equal); writing – review and editing (lead). **Rachael Lappan:** Writing – original draft (equal); writing – review and editing (supporting). **Marcela Hernandez:** Writing – review and editing (supporting). **Talitha Santini:** Writing – review and editing (supporting). **Andrew Tomkins:** Writing – review and editing (supporting). **Chris Greening:** Conceptualization (lead); writing – original draft (equal); writing – review and editing (equal).

## CONFLICT OF INTEREST

The authors declare that they have no conflict of interest. Invited article for Environmental Microbiology (Crystal Ball Special Issue).

## Data Availability

Due to the nature of this research, data sharing is not applicable.
